# A mixed methods approach to obtaining health care provider feedback for the development of a Canadian pediatric dental caries risk assessment tool for children <6 years

**DOI:** 10.3389/froh.2023.1074621

**Published:** 2023-03-29

**Authors:** Robert J. Schroth, Grace Kyoon-Achan, Josh Levesque, Melina Sturym, Daniella DeMaré, Betty-Anne Mittermuller, Juyoung Lee, Victor Ho Kong Lee

**Affiliations:** ^1^ Department of Preventive Dental Science, Dr. Gerald Niznick College of Dentistry, Rady Faculty of Health Sciences, University of Manitoba, Winnipeg, MB, Canada; ^2^ Department of Pediatrics and Child Health, Max Rady College of Medicine, Rady Faculty of Health Sciences, University of Manitoba, Winnipeg, MB, Canada; ^3^ Children’s Hospital Research Institute of Manitoba, Winnipeg, MB, Canada; ^4^ Shared Health Inc., Winnipeg, MB, Canada

**Keywords:** caries risk assessment, early childhood caries (ECC), non-dental primary health care providers, early childhood oral health, focus groups, primary care

## Abstract

**Introduction:**

Early childhood caries (ECC) is a chronic but preventable disease affecting young children worldwide. Many young children face access to care barriers to early preventive dental visits for a variety of reasons, which can increase their risk for ECC. Non-dental primary health care providers are well positioned to assist in assessing a child's risk for ECC by performing caries risk assessment (CRA). The purpose of this project was to report on primary health care provider and stakeholder feedback in order to refine a drafted CRA tool for Canadian children <6 years of age intended for use by non-dental primary health care providers.

**Methods:**

In this mixed methods project, we conducted six focus groups with primarily non-dental primary health care providers followed by a short paper-based survey to quantify preferences and feedback. Data were thematically and descriptively analyzed.

**Results:**

Participants’ feedback on the drafted CRA tool included the need for it to be relatively quick to complete, easy and practical to score, easy to implement into practitioners’ clinic schedules, and to include anticipatory guidance information to share with parents and caregivers. All participants (100%) welcomed a CRA tool. Many (85.4%) liked a layout that could be added to tools they already utilize. Most (73.2%) wanted the tool to be in colour, and many (90.2%) wanted the tool to include pictures.

**Conclusion:**

Non-dental primary health care providers informed the final development and layout of the newly released Canadian CRA tool. Their feedback resulted in a user-friendly CRA tool with provider-patient dynamics and preferences.

## Introduction

There is growing recognition of the need and benefits of integrating oral health into primary care as a means of identifying populations at risk for oral health disparities, and to address access to oral health care challenges. Dental caries is one of the most common chronic diseases in children and adolescents, even though it is largely preventable. Although prevalence of dental caries in the majority of populations has decreased in recent decades, the prevalence of dental caries in preschool-aged children (ages 2–5) has increased in North America (1, 2). This demonstrates that preschool-aged children are at higher risk for oral health disparities, likely caused by barriers in access to oral health care (2).

The rate of pediatric dental surgery under general anesthesia to treat severe early childhood caries (S-ECC) in Manitoba, Canada has increased significantly between 1997 and 2007 (3). Treatment of S-ECC is now the most common and cost intensive day surgical procedure in Canada (4, 5). Treatment under general anesthesia does not prevent future caries development, since it does not treat the underlying causative factors of caries development. In order to decrease the prevalence of caries, prevention needs to be emphasized. It has been determined that non-dental primary health care providers can be significant contributors to caries prevention in young children (6).

Non-dental primary health care providers, such as physicians, nurses, and nurse practitioners, are uniquely well positioned to promote prevention of caries in young children, since they often see children and their caregivers numerous times throughout the early years. This yields a unique opportunity to (1) promote behaviours that reduce the risk for oral health diseases, (2) disseminate oral health and hygiene information, such as discussing the risk of transmission of cariogenic bacteria from caregiver to child, (3) assess risk for caries development, and (4) deliver basic preventive oral health care, such as the application of fluoride varnish. A key to effective prevention is ensuring that parents and caregivers have sufficient knowledge and guidance to take care of their children's teeth. Since non-dental primary health care providers are not experts on oral health, they do not have the ability to manage all oral health care needs. However, they do have the ability to refer children identified as being at high-risk for caries development to dental health care providers in order to establish a dental home before irreversible damage occurs to primary dentition.

Caries risk assessment (CRA) is the determination of the likelihood of the incidence of caries, or the likelihood that there will be a change in the size or activity of existing lesions during a certain time period (7). Caries risk-based care is unique to the traditional restorative approach to dentistry since there is greater emphasis on prevention (8). Additionally, CRA can be performed by non-dental primary health care providers to identify and refer high-risk children before caries development or progression occurs (7). In conjunction to CRA, non-dental primary health care providers may also choose to provide oral hygiene instruction, anticipatory guidance, or basic preventive oral health care treatment.

CRA tools can serve as a useful guide when interviewing children's parents or caregivers with the goal of obtaining key information about a child's past and current behaviours that are known to be significant factors in caries development. By taking a variety of these factors into consideration, children can be categorized as being at low or high-risk for caries development, and be referred as needed.

A systematic review of CRA was conducted to develop an evidence-based Canadian CRA tool to be used for children <6 years of age in non-dental clinical settings (9). Numerous sociodemographic, behavioural, and clinical factors were investigated and selected to be included in the CRA tool based on reports of clinical association to caries risk. Some risk factors and protective factors that were selected to be included in the CRA tool were socioeconomic status, frequency of tooth brushing by a parent, exposure to fluoride, feeding practices, visible dental plaque, and evidence of past or present caries. It has been determined that evidence of past or present caries is the factor most strongly correlated to longitudinal caries outcomes (10). Consequently, for the tool to lead to a meaningful insight into caries risk, this factor was weighted the highest in the scoring system developed for the CRA tool.

In this project, mock-ups of the CRA tool went through pilot testing for feedback from primarily non-dental primary health care providers. The purpose was to obtain and report both quantitative and qualitative feedback from primary health care providers and stakeholders on a drafted CRA tool for Canadian children <6 years of age. The intent was to refine the tool for use by non-dental primary health care providers.

## Methods

Our research team was contracted by the Office of the Chief Dental Officer (OCDO) of Canada at the Public Health Agency of Canada (PHAC) to develop a CRA tool for children <6 years of age for use by non-dental primary health care providers and dental providers in non-dental clinical settings (9, 11, 12). This was a mixed methods project employing the Triangulation Design with specific qualitative and survey methods (13). Focus groups are appropriate for such applied studies (14). We undertook focus group testing with 63 participants to help refine the tool. These participants were stakeholders and primarily non-dental primary health care providers that expressed interest in the project following our queries (invitations) to various groups, agencies, and clinics. This project sought practical feedback to help improve the CRA tool. Simple one-time cross-sectional survey of focus group participants were used to quickly gather quantitative data (15).

Focus groups were conducted with non-dental primary health care providers during the summer and fall of 2018. Five focus groups were held in Winnipeg in September and October with 49 participants. The first focus group had 10 participants: three public health nurses, two dieticians, one pediatrician, one family physician, one speech pathologist, and two dentists. The second focus group had 10 participants: five public health nurses, one pediatrician, one social worker, one public relations administrator for the Manitoba Dental Association, one healthy living facilitator, and one registered dental hygienist. The third focus group had 9 participants: four dieticians, one social worker, one educational assistant, and two office administrators from the Healthy Start for Mom and Me program. The fourth focus group had 11 participants: five nurses, one nurse practitioner, four family physicians, and one dentist from Mount Carmel Clinic. The fifth focus group had nine participants: one nurse, six family physicians, one family physician resident, and one clinic manager from the Northern Connections Medical Centre. A sixth focus group, exclusively with 13 pediatricians and one pediatric resident, all members of the Manitoba Pediatric Society, was held on 8 November 2018 at a clinic in Winnipeg, Manitoba.

These meetings were audio-taped and transcribed for analysis. Members of the project team also took written notes. There were 13 focus group questions that invited participants to comment on various aspects of a draft CRA tool and provide overall impressions of the draft. See Table 1. Data were initially manually clustered, then uploaded to NVivo™ software for further thematic coding and analysis. As this was a quality improvement activity, no ethics approval was required by the University of Manitoba's Health Research Ethics Board. Participants received a twenty-five dollar gift card as an honorarium for taking part in the focus group session.

**Table 1:** Questions asked to health care providers about caries risk assessment tool during focus group sessions held in Winnipeg throughout 2018.

**Focus group questions:**
1. What would make an assessment tool easy for you to use? How much time would you normally be able to spend doing the assessment and applying fluoride varnish?
2. What else would make the overall layout easy to follow?
3. Would you prefer the tool in colour or black and white?
4. Do you find the pictures in versions C1 and C2 helpful? Do you think pictures should be added to the final version?
5. What font style and font size would make the document easier to read?
6. How would you change the layout to make it better?
7. What do you think of the wording of each question? Is there anything you would change?
8. What else is missing that we should be asking about? Do you think anything should be added or taken out from the list of questions?
9. What else would make the follow-up/caries management instructions clearer? Are the current instructions clear? Are they sufficient? Do you think anything should be added or taken out?
10. How many checks on the “high risk” column should equal an overall high caries risk status?
11. Have you ever used any other caries risk assessment tools? Which ones? What did you like about them? What did you not like?
12. In what format would you prefer the assessment tool? Paper, electronic, or both?
13. What name do you prefer?

Participants were also asked to complete a short survey that was administered following the focus group sessions. Participants were asked to complete a short paper-based survey with questions similar to those they had responded to during the focus groups. This was done to quantify feedback and tally points of agreement, predominant preferences, and recommendations for changes to the draft CRA tool. Survey data were entered and descriptively analyzed using the Number Cruncher Statistical Systems (NCSS™) software (Kaysville, Utah).

## Results

There were 63 participants that participated in focus group activities. Forty-nine individuals participated in focus groups 1–5 and 14 participated in the sixth. Focus group participants included physicians, nurses, a nurse practitioner, dentists, a dental hygienist, dieticians, social workers, a speech language pathologist, a healthy living facilitator, a public and media relations director, an educational assistant, and an outreach worker.

### Focus group results

Data were coded to generate overarching themes. The themes represent items that participants pointed out as being important to support non-dental primary health care providers’ use of the CRA tool. Seven themes emerged: (1) the CRA tool needs to be relatively quick to complete, (2) it needs to be easy and practical to score, (3) it needs to be easy to implement into practitioners’ clinic schedules, (4) the instructions and prompts need to be clear, (5) it needs to include anticipatory guidance information to share with parents and caregivers, (6) it must use straightforward language, and (7) it needs to be easy to use. These themes are presented with supporting quotes:

1. It needs to be relatively quick to complete:

Participants wanted to know how much time would be involved in using the tool and possibly conducting basic preventive treatments, such as fluoride varnish application.

*“5–10 minutes would be reasonable/realistic, especially in a non-clinical setting.”*

*“A dental assessment would be 1–2 minutes – applying varnish may be possible in that visit or may require second visit.”*

*“Under 5 minutes to read (non-clinical setting), 5–7 questions (concise, more than enough to support clinical indications).”*

*“Fee-for-service doctors may struggle to use the tool and apply varnish during a scheduled appointment.”*

*“Doctors/nurses have a lot to go through with parents/caregivers during immunization visits and doing [the tool] might be overwhelming for the parent, child, and provider to add something else to these visits.”*

Participants wanted to spend little, but meaningful time applying the tool as they would be incorporating the tool as extra, unpaid activity within their scheduled appointments with patients and clients.

2. It needs to be easy and practical to score:

Participants wanted the information gathered from the tool to contribute to an overall score determining a meaningful caries risk status.

*“Having a total score for the tool.”*

*“A way of ‘totaling a score’ and then determine next steps or pathway for patient.”*

*“Helpful guide for completion on the backside of the page (how many ‘yes’ checks for high risk, what score would make you go from ‘low’ to ‘high’).”*

*“Requires more research. Assuming each factor has different levels of risk, they would have different weight values. If this were done online, with pre-loaded calculations, it would be easier to come up with a score of high or low. Not all settings, however, will have the ability to complete online.”*

*“Good if it could be made clickable (online) chooses/calculates overall caries risk for you and takes you directly to recommendations.”*

Several participants suggested that an online tool that calculates the risk based on a series of indicators would be helpful.

3. It needs to be easy to implement into practitioners’ clinic schedules:

Participants discussed scheduling as an important consideration for seeing patients/clients and applying the tool.

*“Might be challenging depending on how many patients are booked-in in a day.”*

*“CRA tool might work better during visits where no immunizations take place. Probably the 9-month or 15-month visits, which are optional.”*

*“Dental assessment part of this screening, but might not have time for varnish. If more is added to this visit, there is a risk things might be skimmed over.”*

*“Could create a ‘first tooth’ visit regardless of when the first tooth erupts. Can ask parents to bring child in for dental education and then more thorough exam/CRA/varnish during 2-year visit*.*”*

*“Parents could be told in advance about the option of doing a fluoride varnish application during these (optional) visits…will be more prepared mentally and can also prepare the child. Comes down to expectations. If parents know their child is at high risk from a young age, and varnish is something that has to be done at one of the visits, they will do it.”*

Participants said that scheduling opportune times to permit use of the tool and application of fluoride varnish would be ideal.

4. The instructions and prompts need to be clear:

Participants emphasized the importance of words, terms, and concepts being clear for non-dental primary health care providers’ understanding and use.

*“Term ‘brushed’ might be limiting, since some parents may be using a washcloth to clean their children’s gums/teeth, especially when they are very young.”*

*“If only one of the above is a ‘yes’, does that make them a high risk?”*

*“All toddlers are frequent snackers. The issue is what kind of snacks they are eating, drinking after brushing teeth, or not brushing teeth before bed. It has to do with whether children are getting their mouth cleaned after food/drinks. Emphasize parent education.”*

*“What about children who do not drink milk at all? What about benefits of vitamin D and calcium, how that affects dental health. Should that be included in questionnaire?”*

*“Put on guideline page what constitutes a good oral health care routine.”*

*“This question has examples but when you tick off yes or no, should be clearer. They might be yes for one example but not all and it is a bit confusing. Instead of i.e. use e.g.”*

*“Not all tap water is fluoridated, can be misleading to have ‘e.g., tap water’ in statement.”*

Participants requested additional clarity to enable accuracy of oral health messaging, diagnosis, and preventive therapeutic treatment.

5. It needs to include anticipatory guidance information to share with parents and caregivers:

Participants said that they would need more information and education, as using the tool could place them in a position to provide information to patients and clients.

*“Would need to spend some time on oral health education during the appointment – some concern that some parents may be reluctant to have their child get dental care in a primary care setting.”*

*“It would be good to be able see previous responses in the document (when using the form to re-assess the same patient).”*

*“Provide a handout for provider with information about fluoride: When is it applied? How often? How is it applied? Which teeth? How many teeth? What age?”*

*“Some might not exactly know what ‘actions’ to take for each checked ‘yes’ item in the form. Am I supposed to specifically write something regarding the sociodemographic/clinical/protective factor?”*

*“In order for physicians to use the tool, they need to feel like they are well informed on the issues they identify. Handouts for the care providers would be very useful.”*

*“Separate handout to give to parents with recommendations if they do not have fluoridated water. We have many patients that have well water, which I am told does not have fluoride. So is there brief recommendations that can be included in that case?”*

Participants wanted information and education to feel prepared and confident to utilize the CRA tool. Some also wanted information to communicate to parents where necessary.

6. It must use straightforward language:

Participants wanted a French translation, likely in keeping with the bilingual service delivery approach in Canada and more practical wording.

*“French translation.”*

*“’Child’ or ‘pediatric’ should be first in the name for ease of searching in electronic databases.”*

*“Using words that are easy to understand for provider and parent.”*

*“Wording depends on whether you’re going to hand form for parent to fill out or care provider is going to fill it out.”*

*“Care provider can change wording when asking the question. For the most part, primary care providers are asking the questions but the literacy level of parents may be an issue if you give the parents the form to fill out.”*

*“Do we need to say ‘top front teeth’? Could be any teeth. Might be best to say ‘any visible teeth’.”*

*“Not too clinical – we are not dentists. If there are any major concerns we will send [the patients] to [dental professionals].”*

They also wanted use of lay language for parents who may not understand professional jargon.

7. It needs to be easy to use:

Participants said they want a tool that would be easy to use. Comments highlighted the need for legible font type and size, use of pictorial representations making it easy to compare and identify caries, and the overall layout.

a. Font type and size:
  *“Font is a bit small, increase slightly. Need a minimum 10 Arial – standard practice on health information.”*

*“Use black font for headings instead of white.”*

*“Make sure the overall caries risk stands out (larger font, boxed). Be consistent with bullets, bolded sections, etc.”*

*“Having educational information next to the accompanying pictures would be useful for patient education. Action column with resources in it (e.g., Manitoba content like Lift the Lip video, and other educational resources)”*

b. Pictures and colours:
  *“Is there a way to rename or hide high/low risk might deter parents from answering honestly – replace with symbols?”*

*“Add pics of areas where teeth have been extracted. It would be good for education to show parents the consequences of tooth decay; show different pictures, maybe crowns on front teeth. Pictures could be easily integrated into an app.”*

*“Would be nice when sending package to offices, to have colour pictures printed separately from the assessment tool and laminated, so they could be put up in the offices.”*

c. Document layout:
  *“Like the table format of Layout 4, but with pictures on the back, like the breakdown of various questions. Preferred order of factors: SES, protective factors and clinical factors.”*

*“Family history first, and then you can do clinical exam at the end. Clinical questions last makes it less intimidating for both the care provider and the patient. Under ‘caries management’, high risk column before low.”*

It was said that including pictures with the tool could support interaction and explanation of the assessments and follow action. The overall layout may also assist in conversation with parents.

### Survey results

Focus group participants were asked to complete a short survey following their session. Forty-five participants in total completed the survey, yielding a 71.4% response rate. Out of the 45 survey respondents, 37.8% were physicians, 22.2% were public health nurses, and 13.3% were registered dieticians. A complete breakdown of survey respondents’ backgrounds is shown in Table 2.

**Table 2:** Survey respondents’ professional backgrounds.

Professional background	Number of respondents	Percentage (%) of respondents (*n* = 45)
Family doctor (MD)	11	24.4
Pediatrician (MD)	5	11.1
Resident—family medicine (MD)	1	2.2
Nurse practitioner	1	2.2
Nurse (public health)	10	22.2
Dentist	3	6.7
Registered dental hygienist	1	2.2
Registered dietician	6	13.3
Social worker	2	4.4
Speech language pathologist	1	2.2
Healthy living facilitator	1	2.2
Public and media relations director	1	2.2
Educational assistant	1	2.2
Outreach worker	1	2.2

Out of the 49 participants from focus groups 1–5, 41 (83.7%) completed the survey. Most respondents (85.4%) preferred the portrait orientation in order for the tool to be in line with documents in the provincial repository and for inclusion in medical charts. More than half (58.5%) preferred the tool with an “actions” or “considerations” column. Many (73.2%) wanted the tool to be in colour as opposed to black and white. The majority wanted the tool in a format that would be user-friendly for parents and caregivers, specifically having more information that is pictorial (90.2%). Others wanted a visually appealing and easy to use tool. Many (85.4%) said instructions on the tool were clear and sufficient (75.6%). However, the majority still wanted it further simplified with less words. Many (87.8%) had used other CRA tools and most (75.6%) said they would prefer both electronic and paper-based tools.

Out of the 14 participants from the sixth focus group, only four (28.6%) completed the survey. All of those who did respond preferred the tool in portrait layout. It was unanimous that the tool was preferred to be in colour, with pictures added, and that the font type and size was legible with clear caries management instructions. All agreed that the caries management instructions were sufficient, and none had used other CRA tools in the past. All preferred to have electronic or both electronic and paper versions of the tool. The full survey results can be seen in Table 3.

**Table 3:** Survey respondents’ preferences and opinions toward various attributes of the caries risk assessment tool.

Survey respondents’ preferences and opinions	Number of respondents	Percentage (%) of respondents (*n* = 45)
Landscape orientation	5	11.1
Portrait orientation	39	86.7
Indifferent towards orientation	1	2.2
Include “actions” column	24	58.5
Do not include “actions” column	17	41.5
Colour version	34	75.6
Black and white version	10	22.2
Indifferent towards colour	1	2.2
C1—Larger pictures/pictures separate from text	22	53.6
C2—Pictures corresponding to text/more straightforward	15	36.6
D1 (only two focus groups saw this format)	4	22.2
Pictures included	41	91.1
Pictures not included	4	8.9
Font size/style is easy to read	43	95.6
Font size/style is not easy to read	2	4.4
Layout 1	2	4.9
Layout 2	9	21.9
Layout 3	4	9.8
Layout 4	16	39.0
Layout 5	8	19.5
N/A	2	4.9
Follow-up/caries management instructions are clear	39	86.7
Follow-up/caries management instructions are not clear	6	13.3
Follow-up/caries management instructions are sufficient	35	77.8
Follow-up/caries management instructions are not sufficient	10	22.2
Has used a caries risk assessment tool before	5	11.1
Has not used a caries risk assessment tool before	40	88.9
Electronic format	10	22.2
Paper format	1	2.2
Wanted both formats to be available	34	75.6
Name the tool Canadian Pediatric Caries Risk Assessment Tool (<6 years) (CANA-P)	8	19.5
Name the tool Pediatric Canadian Caries Risk Assessment Tool (<6 years) (PE-CAN)	5	12.2
Name the tool Canadian Caries Risk Assessment Tool (<6 years) (CAN-CART)	16	39.0
Do not include “caries” in the name	6	14.8
Name the tool C-CRAT/C-DART	2	4.9
Name the tool Canadian Dental Feedback Tool	1	2.4
Name the tool Dental Health Questionnaire for Kids	1	2.4
Name the tool Canadian Dental Health Tool Questionnaire	1	2.4
EMR Search under Children or Pediatric	1	2.4

Survey question not included in surveys sent to participants of focus group 6.

## Discussion

**Figure 1:**
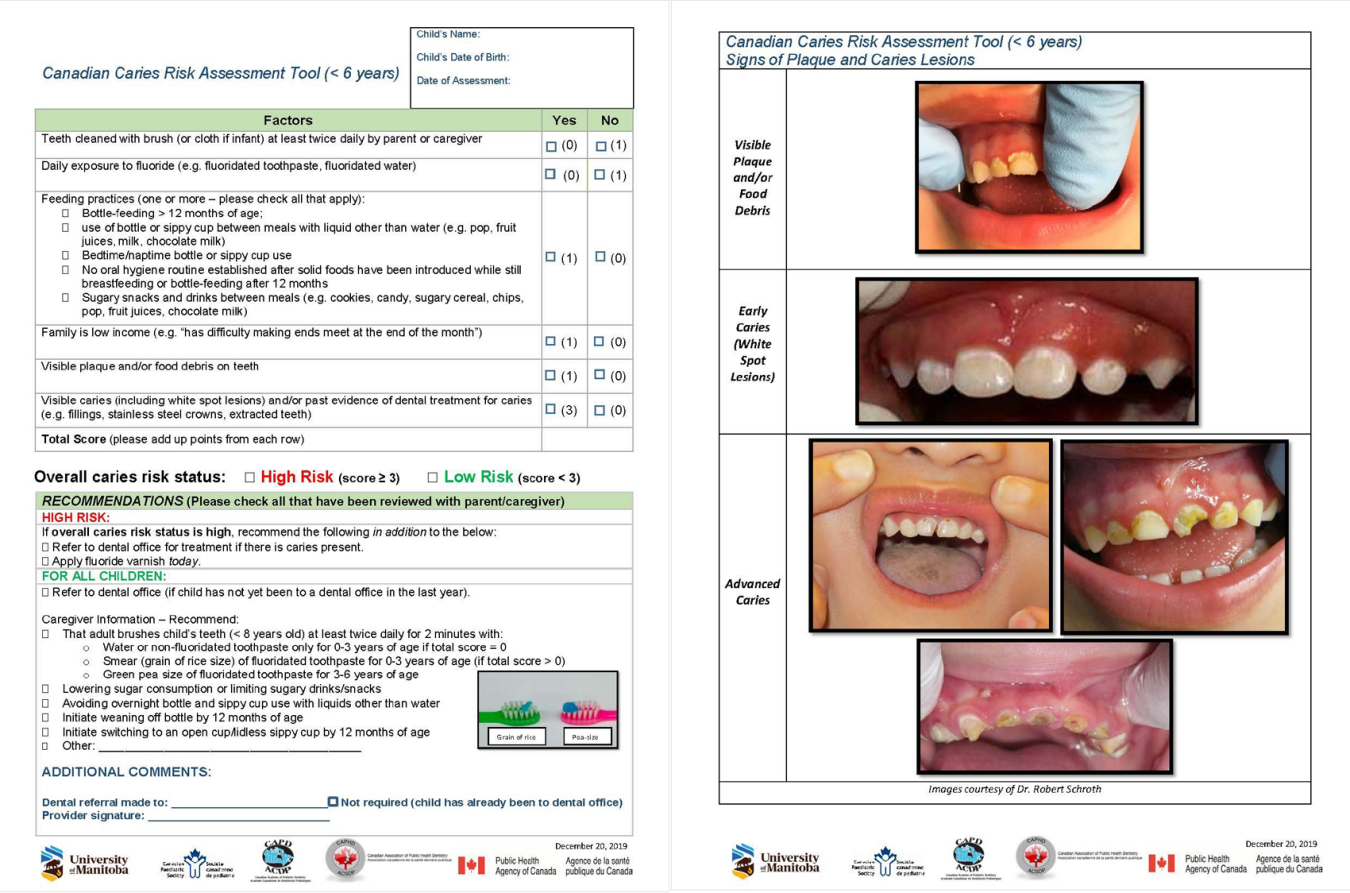
Final version of the Canadian caries risk assessment (CRA) tool available for download (English: http://umanitoba.ca/CRA_Tool_ENG_Version.pdf; French: https://umanitoba.ca/CRA_Tool_FR_version_with_logos.pdf).

Focus group participants provided invaluable feedback during the refinement of the drafted Canadian CRA tool. They expressed the need for the tool to be relatively quick to complete, easy and practical to score, easy to implement into practitioners’ clinic schedules, and to include anticipatory guidance information to share with parents and caregivers. The majority preferred the CRA tool to be in portrait orientation so it would be in line with documents in the provincial repository and for inclusion in medical charts. Most preferred the CRA tool to be in colour and to include pictorial information with the aim of being user-friendly for parents and caregivers. Feedback from participants was used, and relevant changes were made to the CRA tool. This resulted in the creation of the final CRA document, which is presented as Figure 1.

Canadian children can greatly benefit from a collaborative and concerted effort to prevent ECC. Non-dental primary health care providers require some guidance to identify and assess future risk for the disease (16, 17). Dental and non-dental health care providers can apply baseline risk categories in predicting caries existence or progression and apply basic prevention treatments to arrest early onset of caries or make referrals to dentists for additional disease management. It has been shown that health care provider risk assessments accurately predict ECC progression (10, 18). Some non-dental primary health care providers might even conduct basic therapeutic/preventive treatments such as applying fluoride varnish with necessary supports (19).

Focus groups and surveys with non-dental participants in this project indicated openness and willingness to support early childhood oral health (ECOH) by applying a CRA tool to help identify and prevent ECC in young children. This is not surprising as primary care practitioners see patients earlier on and more frequently and can easily incorporate a CRA tool. Some of these practitioners routinely and often look in the oral cavity but are not usually looking for dental disease *per se* (20). CRA are promising tools helping to create standardized measures for determining caries risk that can be used for early disease detection (21, 22).

We have, however, learned that it is crucial that the CRA tool be familiar and in line with similar documents normally used by health care providers. For example, there was a strong preference for the portrait layout to be in line with documents in the provincial repository and for inclusion in medical charts. It should be user friendly, easy to understand with clear and sufficient instructions (23, 24). Information that is evaluative and engages patients have higher chances of adoption and encouraging behaviour change (25). Thus, many would-be users of the CRA tool want it to be in colour versus black and white, in a format that would be user friendly for parents and caregivers, would include information that is pictorial, visually appealing, easy to understand and use.

A growing preference for online and electronic applications in health care is supported by this project (26–30). Electronic applications can be used to build patient history, document progress, support decision-making, and for quality improvement activities (31). Additionally, they can be used to aid in provider-patient communication and joint decision making (32). However, the apps have to be informed by health professionals, with good design detail, grounded and validated for use, as the Canadian CRA tool has been (33, 34).

Although there have been numerous studies that have investigated caries risk factors and the predictive validity of CRA tools, this paper appears to be the first to report on the qualitative analysis of feedback acquired through pilot testing of a CRA tool indicated for use by non-dental primary health care providers (9, 25, 35–49). A similar British study has reported on feedback from the pilot testing of an online integrated oral health and risk assessment tool (DEPPA). However, its use is intended for dental health care providers (50). While this project appears to be the first to report on the qualitative analysis of feedback acquired during the development of a CRA tool, other studies have expressed similar preferences and characteristics of an ideal CRA tool, such as the need for it to be quick to complete, easy to score, and to have clear cutoffs for risk levels to aid in the referral of high-risk patients to dental health care providers (38, 42). The findings of this project are congruent with these preferences and should find to be useful for the development of CRA tools to come.

The OCDO, CPS, Canadian Academy of Pediatric Dentistry, and Canadian Association of Public Health Dentistry have all endorsed this tool. The tool has been added to the online Rourke Baby Record© and is undergoing a pilot validation funded by the Network for Canadian Oral Health Research. Unfortunately, the release of the CRA tool coincided with the first wave of the COVID-19 pandemic, therefore implementation has been delayed. Consequently, service providers may need to be reminded of the tool and how to incorporate it into their clinical practice. We are currently moving to implement use of the CRA tool by training non-dental primary care providers on how to use the tool in some Indigenous communities.

### Strengths and limitations

A strength of this project is that the participants involved were all those who the tool was being designed for. This, in combination with the qualitative research methods employed, resulted in very attentive groups that offered lots of relevant and careful feedback, aiding to make the developed tool be more practical for use (Figure 1).

Contrarily, a limitation of this project is the possibility of sampling bias. Our participants may not have been representative of primary care providers working with children, as only those who were supportive of CRA and our objectives responded to our invitations. Another limitation is that it did not specifically inquire about what barriers practitioners may envisage in applying the tool in their practice. However, this may come up during the upcoming training of non-dental primary care providers in select Indigenous communities. Applications of other tools have returned feedback on barriers hampering uptake, having to do with policy changes, medical practitioner training, and office supports (51). Future research can focus on understanding some of the barriers and challenges faced in implementing the tool since it is validated and available for use by dental and non-dental primary health care providers alike.

## Conclusion

A Canadian CRA tool for use in children <6 years of age was refined through application of feedback obtained from focus groups consisting of non-dental primary health care providers. The result was a user-friendly CRA tool informed for practical use. Beyond informing the final development and layout of the CRA tool, non-dental primary health care provider feedback has also set the stage for future collaborations to prevent ECC and promote ECOH.

## Data Availability

The raw data supporting the conclusions of this article will be made available by the authors, without undue reservation.
